# Clinical Characteristics, Treatment Considerations, and Outcomes of Infants with Rhabdomyosarcoma

**DOI:** 10.3390/cancers15082296

**Published:** 2023-04-14

**Authors:** Adam P. Yan, Rajkumar Venkatramani, Julie A. Bradley, Timothy B. Lautz, Cristian I. Urla, Johannes H. M. Merks, Sapna Oberoi

**Affiliations:** 1 Division of Pediatric Hematology Oncology, The Hospital for Sick Children, University of Toronto, Toronto, ON M5S 1R1, Canada; adam.yan@sickkids.ca; 2 Dana-Farber/Boston Children’s Cancer and Blood Disorders Center and Department of Pediatrics, Harvard Medical School, Boston, MA 02115, USA; 3 Department of Pediatrics, Texas Children’s Hospital, Baylor College of Medicine, Houston, TX 77030, USA; 4 Department of Radiation Oncology, University of Florida, Jacksonville, FL 33024, USA; 5 Department of Surgery, Ann & Robert H Lurie Children’s Hospital of Chicago, Northwestern University, Chicago, IL 60208, USA; 6 Department of Pediatric Surgery and Pediatric Urology, University Children’s Hospital of Tuebingen, Hoppe-Seyler-Strasse 3, 72076 Tuebingen, Germany; 7 Princess Ma’xima Center for Paediatric Oncology, 3584 CS Utrecht, The Netherlands; 8 Division of Imaging and Oncology, University Medical Center Utrecht, Utrecht University, 3584 CS Utrecht, The Netherlands; 9 Department of Pediatrics and Child Health, University of Manitoba, Winnipeg, MB R3T 0A1, Canada; 10 Department of Pediatric Hematology-Oncology, CancerCare Manitoba, Winnipeg, MB R3E 0V9, Canada

**Keywords:** rhabdomyosarcoma, sarcoma, infant, pediatric

## Abstract

**Simple Summary:**

Rhabdomyosarcoma (RMS) is the most common type of soft tissue sarcoma among infants. The clinical characteristics and biology of RMS among infants are distinct from those of older children with RMS. The management of infants with RMS follows a parallel approach of risk-stratification and treatment with multimodality chemotherapy in combination with surgery and/or radiation. but with a few caveats. Modification of chemotherapy regimens is often needed to reduce the risk of excessive treatment-related morbidity due to developmentally immature organs. Adequate surgical resection is more challenging due to the technical difficulties associated with large tumors arising from critical structures in small-sized patients. Similarly, irradiating developing organs can impair long-term function or form and increase the risk of secondary malignant neoplasms. A few clinical trials conducted by international cooperative groups have addressed the challenges of managing infants with RMS and their outcomes.

**Abstract:**

RMS most commonly presents in children and adolescents, however a subset of tumors are diagnosed in infants under one year of age. Due to the rarity of infant RMS, utilization of different treatment approaches and goals, and small sample sizes, the published studies of infants with RMS have yielded heterogeneous results. In this review, we discuss the outcomes of infants with RMS treated in various clinical trials and the strategies that various international cooperative groups have employed to reduce the morbidity and mortality related to treatment without compromising the overall survival of this population. This review discusses the unique scenarios of diagnosing and managing congenitals or neonatal RMS, spindle cell RMS and relapsed RMS. This review concludes by exploring novel approaches to diagnosis and management of infants with RMS that are currently being studied by various international cooperative groups.

## 1. Introduction

Rhabdomyosarcoma (RMS) is the most common type of pediatric soft tissue sarcoma (STS). While most RMS cases occur in children and adolescents, a subset of these tumors known as infantile RMS occur in children under one year of age, and a subset known as congenital RMS occur among infants under 1 or 2 months of age [1,2,3]. Infantile and congenital RMS are distinct, given these patients’ differing clinical characteristics and outcomes compared to older children with RMS [1,4,5,6,7,8,9,10,11,12]. In addition, infants with RMS have unique management challenges, given the technical difficulties of surgery and increased toxicity from chemotherapy and radiotherapy [2,4,5,6,7,8,9,10,11,12,13]. In this article, we will review the epidemiology, clinical characteristics, management, and outcomes of infants with RMS and will focus on the unique aspects of RMS in infants compared to RMS occurring in older children.

## 2. Epidemiology

The annual incidence of RMS in individuals under 20 years is 4.5 cases per million, making it the most common STS in this age group [2]. RMS represents 3.5% of all cancer diagnoses in children 0–14 years and 2% in children 15–19 years of age. The annual incidence of RMS in infancy is greater than in childhood (6.4 cases/million) [14]; however, RMS in infants represents only 5–11% of all RMS diagnoses, and congenital RMS represents only 0.4–1% of all RMS diagnoses [4,5,6,13]. Among infants with STS, RMS is more common than non-rhabdoymosarcoma soft tissue sarcomas (NRSTS); in a European study of 102 infants with STS, 62.7% had a diagnosis of RMS [4].

## 3. Clinicopathologic Characteristics

The Children’s Oncology Group’s (COG) Intergroup Rhabdomyosarcoma Studies (IRS) and ARST studies, the Italian Cooperative Group (ICG), the International Society of Pediatric Oncology’s (SIOP) Malignant Mesenchymal Tumor (MMT) studies, the Cooperative Weichteilsarkom Studiengruppe (CWS), and the European pediatric Soft tissue sarcoma Study Group’s (EpSSG) RMS2005 studies have reported on the clinical characteristics of infants with RMS (Table 1) [1,4,7,8,9,11,12].

### 3.1. Histology and Genomic Alterations

Studies have shown that embryonal RMS (ERMS) and alveolar RMS (ARMS) represent 69–87% and 13–31% of RMS diagnoses among infants, respectively. Specifically, the EpSSG RMS2005 study identified a significantly lower proportion of ARMS among infants than older children [9]. In contrast, data from the ARST studies and the surveillance, epidemiology, and end results (SEER) program did not identify any significant difference in the histologic subtype of RMS between infants and older children [7,11,15]. Botryoid and spindle-cell RMS (SRMS) are also relatively more common among infants than older children [8,10]. SRMS has been reported in 10–22% of infants with RMS [9,16]. A few studies have attempted to evaluate the unique genomic features of infantile RMS. An EpSSG RMS2005 study reported on *FOXO1*-fusion status among infants and found no difference in the proportion of fusion-positive RMS among infants and older children of ages 12–36 months; however, fusion status was not investigated in one-third of the study population [9]. A second study from France determined that all infants aged <6 months with ARMS had *PAX3-FOXO1* fusion [16]. SRMS in older children may be associated with *MYOD1* mutations and has a poor prognosis. In contrast, in infants SRMS is associated with recurring fusions involving *VGLL2* or *NCOA2* genes and carries a favourable prognosis [17,18]. Genomic analysis of a large set of children with RMS treated in North America and the UK identified *RAS* mutations in 64% of infants with RMS. In addition, they found that *FOXO1* fusion translocation-negative tumors in infants frequently harbor *HRAS* mutations (40%) and do not show enrichment of secondary mutations [19].

### 3.2. Primary Tumor Site and Size

The common anatomical sites of infantile RMS include genitourinary (19–45%), extremities (9.7–16%), trunk (9–32%), and non-parameningeal head and neck (8–24%) [4,5,6,7,8,9,10,11,12,15]. The MMT, IRS, and EpSSG studies found fewer parameningeal and orbital tumors among infants than older children [4,5,9]. In contrast, no significant differences in the distribution of tumor sites between infants and older children were identified in the SEER or ARST studies [1,7]. Regarding the distribution of favorable and unfavorable tumor sites, ARST0331 and ARST0531 studies found that infants less than 24 months of age were more likely to have an unfavorable primary site than children older than 24 months (64.5% vs. 54.9%). In comparison, the EpSSG RMS2005 study found no difference in the number of infants with a favorable vs. unfavorable primary site compared to children 12–36 months old [1,7,9]. This difference may be attributable to dissimilarities in the definition of infants by the two studies (24 vs. 12 months), differences in the comparison groups (>24 months vs. 12–36 months) and small sample size [7,9].

Among children with RMS, a primary tumor size of ≥5 cm has been associated with inferior event-free survival (EFS) and overall survival (OS) compared to smaller tumors [6]. Many experts have expressed concern regarding using this metric in infants, as the risk of a tumor ≥5 cm may be different in a 5 Kg infant compared to a 50 Kg adolescent. An Italian study evaluating the impact of patient size and tumor size on the survival outcome of children with localized STS found a significant interaction between size and body surface area (BSA), with mortality increasing from the larger to smaller BSA for a given tumor size [11]. However, a subsequent COG study of children with intermediate-risk RMS, suggested that patient weight did not impact successful tumor resection and did not modify the association between tumor size/volume and EFS [20]. The IRS and EpSSG RMS 2005 studies identified a significant difference in the number of infants presenting with a tumor ≥5 cm compared to older children, whereas this finding was not demonstrated in the CWS and ARST analyses [5,7,9,12].

### 3.3. RMS Predisposing Syndromes

Children with Li-Fraumeni syndrome, *DICER1* syndrome, neurofibromatosis type I, Costello syndrome, Beckwith–Wiedemann syndrome, and Noonan syndrome are all at an increased risk of developing RMS [12,21]. In two studies of children with RMS and Li-Fraumeni syndrome, the age at presentation ranged from 19 to 67 months with median ages of 3.3 and 2.3 years in each of the studies [22,23]. Neither of these studies identified any infants with RMS and Li-Fraumeni syndrome [22,23]. A higher birth weight and large for gestational age infants have also been shown to increase the risk of developing RMS. No data suggest that these children are more likely to present under one year of age than children without these risk factors [24]. Further work is needed to better understand the unique genomic and epigenetic changes that may predispose a child to develop RMS in infancy.

## 4. Approach to Treatment

Effective management of children with RMS requires a multimodal approach consisting of local control with either surgery and/or radiotherapy (RT) and administering a multiagent chemotherapy regimen. The typical approach to managing children with RMS may have to be adjusted when treating infants, given the difficulties of achieving surgical resection in infants and the increased risk of treatment-related morbidity from chemotherapy and radiotherapy among infants compared to older children due to developmental immaturity (Table 2 and Table 3) [4,5,6,7,8,9,10,11,12]. This was apparent in a recently conducted study utilizing the population-level data from the SEER program (2000–2016); the study showed that infants (age < 1 year) were more likely to receive surgery for local control (68.9% vs. 57.5%, *p* = 0.02) and less likely to receive RT (34.0% vs. 66.4%, *p* < 0.001) compared to children aged 1–9 years old with RMS. This study also identified that infants are less likely to receive combined modality treatment, i.e., chemotherapy and local therapy (surgery or RT), compared to older children (75.7 vs. 86.8%) and are more likely to receive treatment with only one therapeutic modality [1]. Significant clinical trial protocol deviations related to omitting or delaying RT for local control among infants with RMS have also been reported in the COG clinical trials, underscoring the concern for toxicity in infants with RMS [7,8]. For instance, in the ARST0331 and ARST0531 COG studies, 43% of infants had deviations from the protocol-specified local control recommendations, most commonly due to omission of RT, and protocol deviations were more common in patients with group III disease compared to those with group I and II disease (51% vs. 30%, *p* = 0.019) [7]. A summary of the studies that reported on the outcome of infants with RMS is presented in Table 4 [4,5,7,8,9,10,11,12,25]. The COG studies have taken a more aggressive approach to manage infant RMS and optimize EFS compared to European cooperative groups’ studies that have used RT more conservatively to minimize long-term morbidity in this population and optimize OS [4,5,7,8,12,25].

### 4.1. Chemotherapy

Chemotherapy regimens used to treat older children with RMS are modified for infants with RMS due to their greater susceptibility to treatment-related toxicity (Table 2) [4,5,6,7,8,9,10,11,12]. Infants are more likely to have significant side effects from chemotherapy compared to older children (treatment-related death in 5% vs. 1%) due to the immaturity of their organs and differences in drug metabolism [11]. In an analysis of chemotherapy-related toxicity from COG’s D9803 protocol, 15% of patients under 36 months developed hepatopathy (vs. 4% for those older than 36 months) [26]. Due to concerns for unacceptable toxicity related to chemotherapy administration, many international cooperative groups decrease the chemotherapy doses given to infants and calculate chemotherapy doses using body weight instead of BSA (Table 4) [4,5,7,8,9,10,11,12,25]. In the MMT 84 and MMT 89 studies, chemotherapy doses were reduced by 50% in infants under 6 months of age and by 33% in infants 6–12 months of age. If the first course of chemotherapy was well tolerated, chemotherapy doses were increased to full doses for the following courses [4]. Despite these initial dose reductions, a second dose reduction occurred in 19–60% of infants receiving chemotherapy in these studies [4]. Similarly, in the IRS studies, unacceptable toxicity from chemotherapy was observed in IRS-I, prompting a reduction in chemotherapy doses in IRS-II and subsequent studies [5,8,10,25]. In the IRS-IV and IRS-V studies, the mean dose of chemotherapy administered per cycle ranged from 58 to 83% for alkylators, 60 to 83% for vincristine, 67 to 89% for dactinomycin, and 58 to 92% for etoposide among infants compared to standard dosing [8]. It is plausible that this reduction in chemotherapy doses may be a contributing factor to the inferior outcomes in the infant population.

### 4.2. Surgical Resection

Due to concerns about the long-term morbidity of RT in infants with RMS, surgery plays an integral role in local therapy [1,12]. Successful surgical resections can be more challenging in infants with RMS than older children [6,10]. Achieving a gross-total resection is paramount when surgery is utilized for local control; debulking procedures provide no survival advantage and do not allow RT dose reduction. Data from multiple studies suggest that achieving an R0 resection is difficult among infants, with reported success rates of 24–56%, with a loss of function, altered cosmesis, or damage to a vital structure occurring in 24–40% of surgeries [7,8,9,12]. Surgical complications are more commonly reported among patients where a complete resection was achieved [8]. Safety and completeness of resection depend on multiple factors, of which the most important are tumor size and tumor location, tumor relationship to adjacent vital structures (vessels, nerves, and organs), as well as the experience and versatility of the surgeon [27]. Generally, surgical principles for local therapy among infants are identical to that for older children with RMS. The upfront surgical resection should not be attempted if the surgery is expected to result in a significant loss of organ function, form, or morbidity. Debulking operations or partial resections must not be attempted due to lack of benefit in survival and added morbidity. For tumors that are not amenable to upfront surgical resection, delayed primary excision (DPE) should be considered after induction chemotherapy. In the COG studies, DPE is generally attempted after 12 weeks of chemotherapy, and in the European studies, after 9 weeks of induction chemotherapy [7,8,9,12]. When R0 resection was achieved either as a result of primary resection and/or DPE, there was a strong association with improved 5-year EFS and OS among infants treated in the CWS studies between 1981 and 2016 [12]. Of note, in these studies, radiotherapy was utilized in <25% of patients, increasing the reliance on chemotherapy and surgical resection. An R0 resection during DPE can allow for a reduction in the radiation dose [7]. Since surgical interventions can be particularly challenging in young infants with large tumors, these patients must be discussed in multidisciplinary tumor boards with surgeons from sarcoma referral centers to ensure that the best surgical approach can be taken with minimal morbidity. Patients requiring complex surgical procedures should have their surgeries performed by experienced surgeons in centers of excellence for the treatment of children with complex solid tumors.

### 4.3. Radiation Therapy

RMS is a radiosensitive tumor, and RT is crucial in achieving local control in these patients. The long-term morbidity from RT can be substantial in infants due to the late effects of RT on developing immature organs. However, RT reduces the risk of local recurrence among infants with RMS. This has been clearly demonstrated by the analysis of children diagnosed with RMS < 24 months treated in ARST0331 and ARST0531 studies [7]. The recommendations for RT were similar for infants and older children in these studies, but local therapy recommendations were not mandated for children < 24 months (Table 3) [4,7,8,9,11,28]. Importantly, in the initial ARST0331 protocol, RT was omitted for group II and III patients with a vaginal primary who had a complete response (CR) to chemotherapy or an R0 section. Given the high rate of local recurrence in this cohort, the protocol was amended to include RT for these patients. Altogether in these trials, 43% of children < 24 months received individualized local treatment, most commonly due to the omission of the RT; the local failure rate was significantly higher among patients that received individualized local therapy than in patients who received protocol-specified local therapy (35% vs. 16%). Delaying local therapy, however, did not significantly impact the cumulative incidence of local failure (26.3% vs. 16.2%, *p* = 0.09) [7]. The IRS-IV and V studies similarly reported 42% of major deviations resulting from protocol-specific RT dose or volume [7].

To avoid the long-term morbidity of RT, the latest CWS studies do not recommend RT for the upfront treatment of infants with RMS if a CR can be achieved with chemotherapy and surgery. Similarly, the RMS2005 study recommended discussion with national coordinating investigators or the multidisciplinary team to decide RT on a case-by-case basis [9,12]. The differences in the philosophies of utilizing RT for local therapy among infants between the North American and European groups are reflected by the higher proportion of the infants receiving RT upfront in the COG ARST0331/0531 studies compared to the MMT-89/95, CWS, and RMS 2005 studies (58% vs. 5% vs. 24% vs. 33.6%) [4,7,9,12]. The 5-year EFS and OS of the infants with the localized disease for infants (<12 months) with RMS2005 (5-year EFS and OS: 73% and 88%) appear comparable to infants (<24 months) treated in the COG ARST studies (5-year EFS and OS: 68% and 82%) [4,7,9,12].

External beam radiation (EBRT) is the most commonly utilized modality for RT among infants, followed by brachytherapy. EBRT can be delivered using photons or protons [29]. Among photon techniques, the COG D9803 study found no difference in 5-year failure of locoregional control (18% vs. 15%) or failure-free survival (FFS) (72% vs. 76%) between patients receiving three-dimensional conformal radiation therapy (3D-CRT) and intensity-modulated radiation therapy (IMRT), respectively [29]. Due to the superior dosimetric properties, reduced integral radiation dose, and elimination of the exit dose beyond the target tumor volume, proton therapy has been increasingly utilized for children with RMS [30]. The dosimetric advantages of protons are particularly valuable for younger children and those with tumors around critical structures such as parameningeal or orbital RMS, for which the target is often close to adjacent organs at risk, creating a risk of significant long-term morbidity. Early outcome data for children with RMS suggest comparable effectiveness of proton and photon RT and promising reductions in toxicity with proton RT, with long-term data still emerging [30,31].

Brachytherapy is another option for radiotherapy modality for infants needing RT (Table 3) [4,7,8,9,11,28]. Brachytherapy was the most common type of RT used for infants in the RMS 2005 study, with 41.7% of infants receiving brachytherapy for local therapy. In this study, brachytherapy was more likely to be used in infants <12 months than older children of 12–36 months [9]. Brachytherapy can provide conformal radiation at a higher dose to the target tissue while sparing adjacent tissues for select primary sites, particularly gynecologic and genitourinary [32,33]. Regarding fractionation, conventional fractionation of 1.8 Gy per fraction remains the modality of choice for delivering RT, as there were no differences in outcomes for patients that received conventional vs. hyper-fractionated RT in the IRS-IV study [25]. Prescription doses have historically ranged from 36 to 50.4 Gy. Radiation prescription dose is determined by stage, group, primary site, and extent of surgical resection. Due to concern for higher local failure rates in patients with larger tumors, ARST1431 evaluated dose escalation to 59.4 Gy for patients with a tumor larger than 5 cm at diagnosis. The results of this approach are pending at this time [34].

## 5. Outcomes

### 5.1. Response to Frontline Therapy and Survival in Localized Disease

Nearly 70–89% of infants with localized RMS have disease control with initial multimodality treatment [4,11,12]. Regarding the likelihood of progression on treatment, both ARST0331/0531 and RMS2005 study analyses found that infants were more likely to progress during treatment than older children (15.3% vs. 5.1%) and (8.3% vs. 5.5%), respectively [7,9]. Historically, studies suggested that the outcome of infants with localized RMS is inferior to older children [11,35]. Contemporary studies suggest no differences in disease control outcomes among infants compared to older children with localized RMS [4,5,6,7,8,9,10,11,12]. For instance, the data from the COG ARST studies demonstrated comparable 5-year EFS and OS between infants (≤24 months) and older children (>24 months), whereas the EpSSG RMS 2005 study identified a superior 5-year OS for infants < 12 months compared to children 12–36 months (88.4% vs. 78%, *p* = 0.020) (Table 4) [4,5,6,7,8,9,10,11,12]. The majority of deaths among infants with RMS are due to the primary disease; however, mortality from treatment toxicity, such as cardiac failure, and secondary malignant neoplasms, such as acute myeloid leukemia, has been reported as well [4,8,11].

### 5.2. Metastatic Disease

A small subset of infants with RMS presents initially with metastatic disease (6–14%) [4,11,12,36]. Most published studies have not separately reported the outcomes of infants with metastatic disease. Of 13 infants with metastatic disease treated in the MMT-84 and MMT-89 studies with an intensified chemotherapy regimen, complete remission was achieved in only three, whereas the rest succumbed to the disease [4]. The inferior survival of infants with metastatic disease was also evident in the CWS infant cohort, where 8 of the 11 patients with the metastatic disease died of disease, 2 had a partial response to treatment, and only 1 patient achieved complete remission [12]. A pooled analysis of data from nine studies performed by the COG and European groups of 788 children with metastatic RMS identified that children under 1 year of age had an inferior EFS compared to children 1 to 9 years of age (25% vs. 36% *p* = <0.0001) [36].

### 5.3. Prognostic Factors

Alveolar histology, IRS group III, tumor size ≥ 5 cm, nodal involvement, and presence of metastases have been identified to unfavorably impact the OS and/or EFS of infants with RMS [4,7,8,11,12]. Not receiving per-protocol local therapy has also been identified as a risk factor for a worse EFS in the COG studies [7,8]. Similarly, in the IRS-IV and V studies, infants who did not receive per-protocol RT had a lower 5-year FFS rate (46% vs. 70%, *p* = 0.075) and significantly inferior 5-year OS (64% vs. 85%, *p* = 0.032) compared to those who did receive RT [8]. In the ARST0331 and ARST0531 studies, for infants (<24 months) who received protocol adherent therapy vs. those who did not receive therapy per the protocol, the 5-year FFS was 77.5% vs. 55.6% (*p* = 0.04), and the 5-year OS was 84.3% vs. 78.5% (*p* = 0.42) [7]. In the ICG studies, local failure was more common for infants for whom the protocol-specified RT was omitted due to patient age (local failure rate of 54% when RT was omitted vs. 28% when RT was received) [11]. The EpSSG RMS2005 study identified an inferior EFS amongst infants that did not receive RT compared to those who did (68% vs. 81%); however, this finding was not statistically significant, and no difference in OS was seen (88% vs. 89%) [9]. On univariate analysis of the CWS cohort, patients who had an R0 resection had an improved 5-year EFS and OS compared to patients with R1/R2 resections, but other factors, including receipt of RT, chemotherapy regimen, response to chemotherapy, IRS group, and primary tumor location, did not impact EFS or OS [12].

### 5.4. Late Therapy-Related Toxicities

Few studies have reported on the long-term morbidity of RMS therapy for infants. Among infants treated in the CWS studies, late treatment toxicities occurred in 85% of patients with a median follow-up of 7.5 years. Significant toxicities included renal toxicity (59%), cardiac toxicity (9%), growth deficiency (9%), and neuropathy (5%). Additionally, 33% of infants undergoing surgical resection of tumors had long-term complications from the surgeries. A total of 6% of surviving infants acquired a second malignancy [12]. Ferrari et al. identified that all six patients who received RT and were alive at follow-up had bone and soft tissue issues [11].

## 6. Infants with Unique Considerations

### 6.1. Congenital/Neonatal RMS

A small subset of infants present with RMS at birth or under 1 month of age; these infants with congenital or neonatal RMS represent 0.4–1% of RMS cases [5,37]. The true incidence may be higher as some of these tumors may be diagnosed after one month of age. Only a few published reports have included specific analyses of congenital RMS; therefore, it is unknown if these patients have unique clinical characteristics and outcomes compared to older infants [5,37,38,39,40]. Most reported cases of neonatal RMS are of embryonal histology; however, alveolar, botryoid, and spindle-cell tumors have also been described in this age group [12]. Of the 24 patients with congenital RMS (diagnosed with RMS during the first two months of birth) in the EpSSG RMS2005 cohort, 23 had favorable histology and localized disease [41]. Neonates with RMS may have unique presenting features, such as the presence of multiple subcutaneous nodules [37,39]. They may also be more likely to present with advanced group III and group IV disease (93% in the CWS infant cohort) [12]. In the EpSSG RMS 2005 cohort, 3 out of 24 patients had *NGLL2-CITED2/NCOA2* fusions [41]. Treatment of neonates with RMS is even more challenging. The MMT84 and MMT89 studies used alternating cycles of single-agent vincristine and dactinomycin for neonates instead of giving both drugs in the same cycle [4]. Other studies have further altered chemotherapy dosing for infants < 3 months of age due to concerns for heightened toxicity related to developmental immaturity of the organs (Table 4) [4,5,7,8,9,10,11,12,25]. The importance of either upfront surgical resection or DPE after chemotherapy in achieving complete remission among patients with congenital RMS (<2 months of age) was demonstrated in the RMS2005 cohort. In this cohort of 24 patients with congenital RMS, 4 of the 5 patients not having surgical resection had progression of the disease [41]. The survival of patients with congenital/neonatal RMS differed among the published studies, likely due to the different definitions of congenital RMS and selection bias. In a retrospective multi-institutional study conducted by the Children’s Cancer Group, only 2 of the 11 neonates with RMS survived [40]. In the CWS studies, although complete remission was achieved in 10 out of 15 neonates, 6 relapsed and died; the 5-year EFS and OS for neonates in this cohort were 20% and 40%, respectively [12]. In the EpSSG RMS2005, the cohort of congenital RMS patients demonstrated an improved 5-year EFS of 75.0% and OS of 87.3% [41].

### 6.2. Congenital Spindle-Cell Rhabdomyosarcoma

SRMS is a rare RMS variant accounting for approximately 10% of RMS cases in infants [11,16]. A recently published international cohort study of 40 infants with SRMS found that 39 had localized disease at the time of presentation, while one patient had metastatic disease; 26 patients in this cohort underwent molecular evaluation, and 50% were found to have *NCOA* and/or *VGLL2* rearrangements. Treatment included chemotherapy (n = 37), surgical resection (n = 31), and RT (n = 5; used following resection with residual microscopic disease). Complete remission was achieved in 95% of the patients. The 5-year EFS and OS for patients with the localized disease were 86% and 91%, respectively, drawing the authors to conclude that with a limited use of RT, most infants with SRMS can be cured with conservative treatment [17].

### 6.3. Risk and Management of Relapsed Disease

The risk of relapse after achieving complete remission with multimodality therapy ranges from 12 to 52% among infants with RMS (Table 4) [4,7,8,9,11,12]. The relapse risk is lower among infants with SRMS (7.8%) [17]. Local failure represents the most common form of relapse (64–85%), followed by distant (8–23%) and combined (5–10%) relapses among infants [4,7,8,9,11,12]. Relapses can occur as early as 3 months and later than 8 years after initial therapy is complete, with a median time to relapse of 1 year [4,7,8,9,11,12]. Regimens utilized for the treatment of older children with relapsed RMS, such as vincristine/irinotecan (VI), vinorelbine/cyclophosphamide/temsirolimus, vincristine/irinotecan/temozolomide (VIT), or temsirolimus/irinotecan can be employed for infants with RMS with relapse based on the initial treatment and type of relapse [42,43]. In the CWS-91-Rez study, 65% of infants with relapsed disease achieved a second complete remission using oral cyclophosphamide and intravenous vinblastine (CYC/VBL) or oral trofosfamide, idarubicin, and etoposide (O-TIE) at relapse [44]. Other studies of combined modality treatment post-relapse have achieved a second complete remission in 23 to 46% of infants with relapsed disease [11,45,46,47]. Age at relapse >1.5 years and time to relapse ≥1 year were identified as significant prognostic factors for five-year progression-free survival (PFS) but not OS after relapse [12]. Of the 51 infants that relapsed in the CWS cohort, the 5-year PFS and OS were 39% and 41%, respectively, compared to 24% and 28% for older children [45,48,49]. This suggests that the outcomes for infants with relapsed disease perhaps are better than that for older children with relapsed disease [21].

## 7. Future Directions

Given the rarity of infant RMS and the heterogeneity of published data, international cooperative groups must compile clinical trial data to generate a sufficient sample size to answer the key questions in optimizing treatment for infants with RMS. To this end, the International Soft Tissue Sarcoma Consortium is working to harmonize data from children with STS treated in the completed COG, CWS, and EpSSG trials and formulate consensus recommendations for the management of children with RMS [34,43]. Future studies should also explore how novel agents can be incorporated into managing infants with RMS. For instance, given the relatively higher proportion of *HRAS* mutations among infants with FN-RMS, future studies may explore using Tipifarnib (farnesyl transferase inhibitor) in this population [19,50]. Another active area of interest is evaluating circulating tumor DNA (ctDNA) at diagnosis and during treatment to tailor therapy based on disease response [51]. Future studies should also examine the long-term functioning and quality of life of patients treated for RMS during infancy.

## 8. Conclusions

RMS occurring under one year of age is a rare entity that may be biologically distinct from RMS in older children. Special considerations in management are required given the heightened risk of chemotherapy-related toxicities and late effects from radiation therapy and the difficulty of obtaining a complete and non-disfiguring surgical resection. Families should be appropriately counselled on the risks and benefits and involved in all aspects of decision-making. Comprehensive molecular analysis is recommended for infants with RMS to identify genomic alterations that may have therapeutic or prognostic implications. Every infant with RMS should be treated on a clinical trial whenever feasible. Further collaborative studies are needed to better define the best treatment approach for this vulnerable population.

## Figures and Tables

**Table 1:** Clinical characteristics of infants with rhabdomyosarcoma.

Characteristic	SEER Data (n = 103) [1]	IRS-IV and IRS-V (n = 76) [8]	MMT 84 and MMT89 (n = 64) [4]	ICG (n = 50) [11]	ARST0331 and 0531 ^a^ (n = 124) [7]	CWS ^b^ (n = 144) [12]	EpSSG RMS 2005 (n = 110) [9]
Sex							
Female	42 (41%)	31 (41%)	22 (33%)	29 (58%)	57 (46%)	61 (42%)	NR
Male	61 (59%)	45 (59%)	44 (66%)	21 (42%)	67 (54%)	83 (58%)	NR
Primary tumor site							
Favorable	NR	29 (38%)	NR	NR	44 (36%)	NR	33 (30%)
Unfavorable	NR	43(57%)	NR	NR	80 (65%)	NR	77 (70%)
Unknown	NR	4 (5%)	NR	NR	0 (0%)	NR	0 (0%)
Specific site							
Genitourinary	20 (19%)	24 (32%)	13 (20%)	22 (44%)	50 (40%)	50 (35%)	50 (46%)
Extremity	10 (10%)	12 (16%)	15 (23%)	10 (20%)	13 (10%)	18 (13%)	15 (14%)
Trunk	33 (32%)	16 (21%)	NR	NR	11 (9%)	NR	NR
Non-parameningeal	24 (23%)	13 (17%)	8 (13%)	28 (56%)	10 (8%)	35 (24%) ^c^	15 (14%)
head/neck							
Parameningal	4 (4%)	3 (4%)	2 (3%)	12 (24%)	17 (14%)	NR	8 (7%)
Orbit	9 (9%)	4 (5%)	4 (6%)	4 (8%)	7 (6%)	8 (6%)	1 (1%)
Other sites	3 (3%)	4 (5%)	10 (16%)	24 (48%)	16 (13%)	33 (23%)	21 (19%)
Histology							
Alveolar	24 (23.3%)	43 (57%)	20 (31%)	11 (22%)	33 (26.6%)	32 (22%)	14 (13%)
Embryonal	79 (76.7%) ^d^	16 (21%)	33 (52%)	37 (74%)	91 (73.4%)	95 (66%)	85 (77.3%)
Botryoid	0 (0%)	0 (0%)	11 (17%)	NR	0 (0%)	10 (7%)	^e^
Spindle cell	0 (0%)	0 (0%)	0 (0%)	0 (0%)	0 (0%)	6 (4%)	11 (10%)
Other/Unknown	0 (0%)	17 (22%)	0 (0%)	2 (4%)	0 (0%)	1 (1%)	0 (0%)
Stage							
Stage 1	NR	29 (38%)	26 (41%)	NR	44 (35.5%)	NR	NR
Stage 2	NR	19 (25%)	25 (39%)	NR	33 (26.6%)	NR	NR
Stage 3	NR	28 (37%)	4 (6%)	NR	47 (37.9%)	NR	NR
Stage 4	NR	(0%)	9 (14%)	NR	0 (0%)	NR	NR
IRS Group							
I	NR	18 (24%)	8 (13%)	5 (10%)	20 (16%)	17 (12%)	3 (3%)
II	NR	15 (20%)	15 (23%)	7 (14%)	25 (20%)	24 (17%)	18 (16%)
III	NR	43 (57%)	32 (50%)	35 (70%)	79 (74%)	103 (72%)	89 (81%)
IV	NR	0 (0%)	9 (14%)	3 (6%)	0 (0%)	0 (0%)	0 (0%)
Tumor classification							
T1	NR	45 (59%)	30 (47%)	24 (48%)	NR	66 (46%)	78 (71%)
T2	NR	31 (41%)	25 (39%)	8 (16%)	NR	73 (51%)	29 (26%)
M	NR	0 (0%)	9 (14%)	3 (6%)	NR	0 (0%)	0 (0%)
Unknown	NR	0 (0%)	(0%)	15 (30%)	NR	0 (0%)	3 (3%)
Tumor size (cm)							
≤5	NR	45 (59%)	NR	6 (12%)	77 (62%)	76 (53%)	63 (57%)
>5	NR	31 (41%)	NR	44 (88%)	45 (36%)	65 (45%)	46 (42%)
Unknown	NR	0 (0%)	NR	0 (0%)	2 (2%)	3 (2%)	0 (0%)
Node status							
N0	NR	63 (84%)	51 (80%)	NR	NR	110 (76%)	109 (94%)
N1	NR	9 (12%)	13 (20%)	NR	NR	14 (10%)	5 (5%)
Unknown	NR	3 (4%)	0 (0%)	NR	NR	20 (14%)	1 (1%)

^a^ Included patients less than 2 years of age with non-metastatic RMS. ^b^ Data for 144 patients with localized disease. However, an additional 11 patients with metastatic disease are discussed separately in the paper. ^c^ Both parameningeal and non-parameningeal head and neck tumors reported together. ^d^ “Other” histology included with embryonal. ^e^ Botryoid tumors included with embryonal.

**Table 2:** Frontline chemotherapy regimens and dose modifications for infants under one year of age with non-metastatic rhabdomyosarcoma in clinical trials.

Cooperative Group	Study	Suggested Chemotherapy	Chemotherapy Modifications
International Rhabdomyosarcoma Study (IRS) [5,6,10]	IRS-IV	All: VAC, VAI or VIE	<12 months: Dose reduced by 50% and escalated as tolerated.
	IRS-V	Low risk: VAC or VI Intermediate risk: VAC or VAC/VTC	
Children’s Oncology Group (COG) [7]	ARST0331	Low risk: VDC	Doses calculated by body weight (mg/kg/dose).
	ARST0531	Intermediate risk: VAC or VAC/VI	Doses calculated by body weight (mg/kg/dose).
Society of Pediatric Oncology (SIOP) [4,12]	MMT84	Group 1: IVA × 3 cycles Group 2–3: IVA × 6–10 cyclesNeonate: Alternating cycles of single agent VA	<6 months: Dose reduced by 50%. Increased to 100% after first cycle if tolerated. 6–12 months: Dose reduced by 33%. Increased to 100% after first cycle if tolerated.
	MMT89	Group 1: VA × 4 cycles Group 2–3: IVA × 6 cycles Neonate: Alternating cycles of single agent VA Metastatic, node-positive or parameningeal: IVA, CEV, & IVE × 6 cycles	
European pediatric Soft tissue sarcoma Study Group (EpSSG) [9]	RMS 2005	VA, IVA or IVADo	<1 month: Ifosfamide and anthracyclines omitted. Ifosfamide added when >1 month and anthracyclines added when >3 months. Other chemotherapy calculated by body weight (mg/kg/dose). 1–3 months: Anthracyclines omitted. Ifosfamide dose calculated by body weight and then reduced to 50%. Other chemotherapy doses calculated by body weight (mg/kg/dose). 3–12 months: Chemotherapy doses calculated by body weight (mg/kg/dose).
Italian Cooperative Group (ICG) [11]	ICG RMS-79	VAC/CAV	<6 months: Dose reduced by 50% and escalated as tolerated. <12 months: Dose calculated by body weight.
	ICG RMS-88	VAIA or IVA	<12 months: Dose calculated by body weight. Dose reduced by 50% and escalated as tolerated.
	ICG RMS-96	CEVAIE	<3 months: No anthracyclines <6 months: All doses reduced by 50% and escalated as tolerated. <12 months: Dose calculated by body weight. Anthracyclines dose reduced by 33% and escalated as tolerated.
	INT	VACA	Dose calculated by body weight. Dose reduced by 33% and escalated as tolerated.
Cooperative Weichteilsarkom Studiengruppe (CWS) [12]	CWS-81	VACA	Dose calculated by body weight.
	CWS-86	VAIA	Dose calculated by body weight.
	CWS-91	VACA or EVAIA	Dose calculated by body weight.
	CWS-96	VA, I2VA or VAIA/CEVAIE	<6 months: 1/3 dose reduction and calculated by body weight. 6–12 months: 1/3 dose reduction and calculated by BSA.
	CWS-2002P	VA, I2VA, or VAIA	<6 months: 1/3 dose reduction and calculated by body weight. 6–12 months (or <10Kg): 1/3 dose reduction and calculated by BSA.
	SoTiSaR	VA, I2VA, or VAIA	<3 months: No ifosfamide or anthracyclines. <6 months (or 10 Kg): 1/3 dose reduction. <12 months: Calculated by body weight.

Abbreviations: CAV: cyclophosphamide, doxorubicin, vincristine, CEV: carboplatin, etoposide, vincristine, CEVAIE: carboplatin, epi-doxorubicin, vincristine, actinomycin, ifosfamide, etoposide, EVAIA: etoposide, vincristine, adriamycin, ifosfamide, actinomycin, I2VA: ifosfamide, vincristine, actinomycin, IVA: ifosfamide, vincristine, actinomycin, IVADo: ifosfamide, vincristine, actinomycin, doxorubicin, IVE: ifosfamide, vincristine, etoposide.

**Table 3:** Radiation therapy recommendations for local control for infants under one year of age with rhabdomyosarcoma in clinical trials.

Trial(s):	Group I	Group II	Group III
IRS-IV & IRS-V [8]	No radiation for ERMS 36 Gy for ARMS and undifferentiated histology	41.4 Gy	Randomized to receive 50.4 Gy (28 × 1.8 Gy fractions) or 59.4 Gy (54 × 1.1 Gy fractions)
MMT 84 & MMT 89 [4]	Radiation therapy reserved for patients without a complete response following primary surgery and/or chemotherapy where a conservative second surgery was not possible. Brachytherapy suggested as a first-line radiation modality when possible.		
Italian Cooperative Group Studies (ICG RMS-79/88/96 & INT) [11]	Infants with unresectable tumors, who did not obtain complete tumor regression with chemotherapy, received 40–45 Gy of conventional radiation, or 32–44 G of hyperfractionated radiation or brachytherapy.		
ARST0331 & ARST0531 [7] ^a^	No radiation for ERMS 36 Gy for ARMS except when the tumor bed no longer existed following surgery	36 Gy for lymph node negative RMS 41.4 Gy for lymph node-positive RMS	50.4 Gy
	For patients undergoing delayed primary excision radiation therapy dosing was adjusted: 36 Gy for complete R0 resection, 36–41.4 Gy for microscopic residual disease (R1 resection), and 50.4 Gy for gross residual disease (R2 resection).		
Cooperative Weichteilsarkom Studiengruppe Studies [12]	No radiation therapy recommended in the CWS-86, CWS-91, CWS-96, CWS-2002P and SoTISaR studies. CWS-81 recommended 48 Gy for Group III tumors and 54.4 Gy for ARMS.		
European pediatric Soft tissue Sarcoma Study Group RMS 2005 [9]	Individualized radiation therapy recommendations based on tumor board discussion with study investigators.		

Abbreviations: ARMS: alveloar rhabomyosarcoma. ^a^ Included patients less than 2 years of age with non-metastatic RMS. The recommendation of radiation for infants did not differ from older children. However, deviations from the protocol-recommended local control radiation guidelines were not considered as protocol violations.

**Table 4:** Summary of studies of localized rhabdomyosarcoma that included infant (<1 year of age) specific analyses.

Cooperative Group	Study	Years	Infants Included ^a^	Infant 5-yr EFS	Infant 5-yr OS	Non-Infant 5-yr EFS ^a^	Non-Infant 5-yr OS ^b^	Overall Relapse Rate	Sites of Relapse		
									Local	Distant	Combined
International Rhabdomyosarcoma Study (IRS) [5,6,10]	IRS-I	1972–1978	78	57%	72%	55%	69%	NR	NR	NR	NR
	IRS-II	1978–1984									
	IRS-III	1984–1991	61	NR	NR	NR	NR	NR	NR	NR	NR
	IRS-IV	1991–1997	41	57%	76%	81%	87%	38%	69%	21%	10%
	IRS-V	1997–2005	35								
Italian Cooperative Group (ICG) [11]	ICG RMS-79	1979–2001	50	62%	42%	NR	NR	52%	85%	8%	8%
	ICG RMS-88										
	ICG RMS-96										
	INT										
Society of Pediatric Oncology (SIOP) ^c^ [4,12]	MMT-84	1984–1995	102	60%	73%	NR	NR	34%	69%	23%	8%
	MMT-89										
Cooperative Weichteilsarkom Studiengruppe (CWS) [12]	CWS-81	1981–2016	155	51%	69%	NR	NR	39%	NR	NR	NR
	CWS-86										
	CWS-91										
	CWS-96										
	CWS-2002P										
	SoTiSaR										
Children’s Oncology Group (COG) [7]	ARST0331	2004–2011	124	68%	82%	NR	NR	31%	64%	23%	5%
	ARST0531	2006–2012									
European pediatric Soft tissue sarcoma Study Group (EpSSG) [9]	RMS 2005	2005–2016	110	73%	88%	68%	78%	12%	69%	8%	23%

Abbreviations: NR: Not Reported. ^a^ ICG and SIOP studies contain metastatic patients in addition to patients with localized disease. Infant is defined as a child < 1 year. The ARST0331 and ARST0531 studies included children < 2 years in the infant analyses. ^b^ Non-infant cohort consisted of patients aged: 1–20 years in IRS-I, ≥1 year (upper limit not specified) in IRS-IV/V, >24 months in ARST studies and 1–3 years in EpSSG RMS2005. ^c^ Analysis also includes infants with non-rhabdomyosarcoma soft tissue sarcomas.
